# IgA1 deposition may induce NLRP3 expression and macrophage transdifferentiation of podocyte in IgA nephropathy

**DOI:** 10.1186/s12967-019-02157-2

**Published:** 2019-12-03

**Authors:** Wei Peng, Gai-qin Pei, Yi Tang, Li Tan, Wei Qin

**Affiliations:** 1 Division of Nephrology, West China Hospital, Sichuan University, Chengdu, China; 2 Division of Nephrology, The First Hospital of Fuzhou City, Fujian Medical University, Fuzhou, China; 3 Department of Nephrology, West China Hospital, Sichuan University, 37th Guoxuexiang Road, Chengdu, 610041 China

**Keywords:** IgA nephropathy, NLRP3, Podocyte macrophage transdifferentiation

## Abstract

**Background:**

The NLRP3 inflammasome plays an important role in mediating podocyte injury in various kidney diseases. The aim of this study was to investigate whether NLRP3 expression associated with podocyte injury was involved in the pathogenesis of IgA nephropathy (IgAN).

**Methods:**

NLRP3 inflammasomes and macrophage marker (F4/80) were detected in the renal tissues of IgAN patients. Association between kidney NLRP3 levels and the clinical feature of IgAN patients was analyzed. Podocytes were incubated with serum containing dys-glycosylated IgA1 protein isolated from IgAN patients. Expression of NLRP3 inflammasomes, F4/80, inflammatory cytokine and renal fibrosis marker were measured using RT-PCR and Western blotting.

**Results:**

Renal NLRP3 inflammasome expression was significantly increased in IgAN patients compared to normal control tissues. Moreover, co-expression of NLRP3 and F4/80 could be observed in the podocytes of IgAN patients. Patients with eGFR < 60 ml/min/1.73 m^2^ had remarkably higher tubular NLRP3 expression (*P *< 0.05), while patients with gross proteinuria (≥ 3.5 g/day) had a significantly higher glomerular NLRP3 expression (*P *< 0.05). Further analysis indicated that dys-glycosylated IgA1 isolated from IgAN patient serum could induce podocyte expression of NLRP3 and the macrophage marker F4/80, which could lead to induction of an inflammatory reaction (increased expression of ICAM-1) and fibrosis (increased expression of α-SMA).

**Conclusion:**

Dys-glycosylated IgA1 isolated from IgAN patient serum could induce NLRP3 expression in podocytes and initiate podocyte macrophage transdifferentiation (PMT). After PMT, podocytes secrete proinflammatory cytokines that can contribute to the inflammation cascade and renal fibrosis changes associated with IgAN.

## Background

Immunoglobin A nephropathy (IgAN) is characterized by the IgA accumulates in the kidneys and is the most prevalent cause of primary glomerulonephritis worldwide [1]. 15–40% of IgAN patients will go on to develop end-stage renal disease (ESRD) within 10–20 years of onset of IgAN [2]. NLRP3 (NOD-like receptor, pyrin domain-containing 3) is an important component of the innate immune system. Previous reports have found that NLRP3 is highly expressed in renal tubular epithelial cells in IgAN and its increased expression is associated with tubular injury. The expression of NLRP3 and activation of its downstream signaling proteins in glomeruli has been described by several studies for various renal diseases [3–6]. In lupus nephritis, the NLRP3 inflammasome was found to be activated in podocytes from biopsy and urine samples, which promoted podocyte injury and proteinuria [4]. Increased NLRP3 inflammasome activity is also implicated in podocyte injury and glomerular sclerosis in hyperhomocysteinemia [7]. Podocyte injury is known to be an important factor for progression of glomerulosclerosis in IgAN [8]. Recent studies have shown that podocytes can acquire macrophage-like functions and activate specific T cell responses (podocyte macrophage transdifferentiation, or PMT), contributing to development of increased inflammation; these findings indicate that podocytes and macrophages may share lineage commitment [9]. Whether this newly described function is relevant to podocytes in the context of IgAN has yet to be explored. Therefore, we conducted this preliminary study to clarify whether activity of the NLRP3 inflammasome or PMT is involved in podocyte injury and pathogenesis in IgAN.

## Methods

### Subjects

Twenty-four patients with primary IgAN confirmed via renal biopsy were recruited from West China Hospital of Sichuan University. Patients with chronic systemic diseases (systemic lupus erythematosus, diabetes mellitus, Henoch–Schönlein purpura, liver cirrhosis, etc.) or advanced renal failure (estimated glomerular filtration rate (eGFR) ≤ 15 ml/min/1.73 m^2^) were excluded. eGFR levels were calculated using the Chronic Kidney Disease Epidemiology Collaboration (CKD-EPI) equation. Clinical information (gender, age, disease history) and laboratory data (serum albumin, serum creatinine, uric acid, 24 h urinary protein and eGFR levels) were collected at the time of biopsy. Histologically normal kidney tissues dissected adjacent to renal tumor were used as controls (n = 8). Written informed consent was obtained from all subjects and the study was approved by the Ethics Committee of West China Hospital of Sichuan University.

### Isolation of serum IgA1

IgA1 was isolated from the serum of IgAN patients using jacalin affinity chromatography (Sigma, Saint Louis, MO, USA) and a Sephacryl S-200 molecular sieve column (GE Healthcare) as previously described [10, 11]. Briefly, jacalin columns were prepared using jacalin-immobilized agarose resin. Serum samples were diluted 1:1 with 175 mM Tris–HCl (pH 7.4), filtered through a 0.2 μm Corning syringe filter, applied to the column, and then washed with 175 mM Tris–HCl (pH 7.4) until the optical density at 280 nm was less than 0.01. Bound IgA1 was then eluted with 0.1 M melibiose in 175 mM Tris–HCl (pH 7.4) until the optical density returned to 0.01. The eluted fractions were pooled, concentrated and applied to a Sephacryl S-200 molecular sieve column. The IgA1 content of the samples was verified by Western blotting and samples were then kept frozen at − 80 °C for future studies.

### Immunofluorescent and immunohistochemical staining

Human kidney tissue sections were analyzed with immunohistochemical and immunofluorescent staining. The sections were deparaffinized and treated with 3% hydrogen dioxide. After antigen retrieval and blocking, sections were incubated overnight with a human NLRP3 antibody (diluted 1:100, R&D Systems), human α-SMA antibody (diluted 1:200, Cell Signaling Technology) or a human ICAM-1 antibody (diluted 1:100, R&D Systems). After treatment with a horseradish peroxidase-labeled biotin-conjugated secondary antibody (diluted 1:200, Biosynthesis Biotechnology, China) and DAB staining (ZSGB-Bio, China), sections were observed under a microscope (ZEISS, Axioimager.Z2). 10 randomly selected fields (400×) were imaged per section. The expression of NLRP3 in glomeruli and tubules was quantified using Image-Pro Plus Image Analysis Software (Meyer Instruments, Inc., Houston, TX, USA); expression of α-SMA and ICAM-1 in glomeruli was quantified in the same manner. The integrated optical density (IOD) was measured for each image by two people independently. The average IOD/positively stained area (AIOD) was then calculated. NLRP3, α-SMA and ICAM-1 expression levels in IgAN renal biopsies and normal kidney biopsies were compared by calculating the AIOD. Double-label immunofluorescence studies were performed with sequential incubation of tissue sections and with different sets of primary antibodies and fluorochrome-conjugated secondary antibodies. Sections were incubated overnight at 4 °C with primary antibodies (anti-NLRP3, 1:100, R&D Systems; anti-podocalyxin, 1:200, R&D Systems; anti-IgA, 1:200, Abcam). After washing, the sections were incubated with the corresponding secondary antibody for 60 min at 37 °C. Nuclei were stained with DAPI at room temperature for 5 min. Images of fluorescently labeled sections were obtained using a fluorescent microscope (ZEISS, Axioimager.Z2). Cultured podocytes were also fixed and stained as described above.

### In vitro IgA1 stimulation of MPC-5 cells

The mouse podocyte cell line MPC-5 was kindly provided by Professor Ping FU. Experiments were performed using low passage (passage 10–18), growth-restricted, conditionally immortalized MPC-5 cells as previously described [11]. To maintain cells in the undifferentiated state, cells were grown under “growth permissive” conditions, which involved growing cells at 33 °C in the presence of IFN-γ (50 U/ml). To induce podocyte differentiation, cells were grown under “restrictive conditions” in the absence of IFN-γ at 37 °C under 5% CO_2_ for more than 12 days. The differentiated cells were incubated overnight in RPMI 1640 containing 0.5% FBS, which was followed by stimulation with or without 0.5 mg/mL serum IgA1 isolated as previously described for 36 h.

### RT-PCR and Western blot analysis

RNA was extracted from cultured cells using TriZOL reagent (Invitrogen) and cDNA was prepared according to the manufacturer’s instructions (Bio-radiScript™ Reverse Transcriptase). Primers used were as follow: mouse GAPDH (forward 5-GCATGGCCTTCCGTGTTC-3; reverse 5-GATGTCATCATACTTGGCAGGTTT-3); mouse NLRP3 (forward 5-AGAGCCTACAGTTGGGTGAAATG-3; reverse 5-CCACGCCTACCAGGAAATCTC-3); mouse α-SMA (forward 5-GTCCCAGACATCAGGGAGTAA-3; reverse 5-TCGGATACTTCAGCGTCAGGA-3); mouse ICAM-1 (forward 5-TCAGGTATCCATCCATCCCAGAGA-3; reverse 5-AGCTCATCTTTCAGCCACTGAGTC-3). RT-PCR was performed as previously described [12]. The expression level of target genes was calculated using the delta-CT method. The supernatants of protein lysates from cultured podocyte were collected for Western blot analysis. The primary antibodies used were mouse NLRP3 (R&D Systems, 1:100), mouse ICAM-1 (R&D Systems, 1:300), mouse α-SMA (Cell Signaling Technology, 1:500), mouse F4/80 (Abcam, 1:50), and mouse β-tubulin (Santa Cruz Biotechnology, 1:1000). Horseradish peroxidase-conjugated rabbit or goat IgG antibodies were used as secondary antibodies (Cell Signaling Technology). Densitometric results were analyzed with Image J software. All protein measurements were normalized to β-tubulin.

### Statistical analyses

Statistical analysis was performed using SPSS 22.0. The data were expressed as mean ± SD or as medians (range). Significant differences were assessed using either at-test or one-way ANOVA. A nonparametric Mann–Whitney U test was performed to compare the integrated optical density between experimental groups. A two-tailed *P *< 0.05 was considered statistically significant.

## Results

### Podocytes express NLRP3 in IgAN

**Fig. 1:**
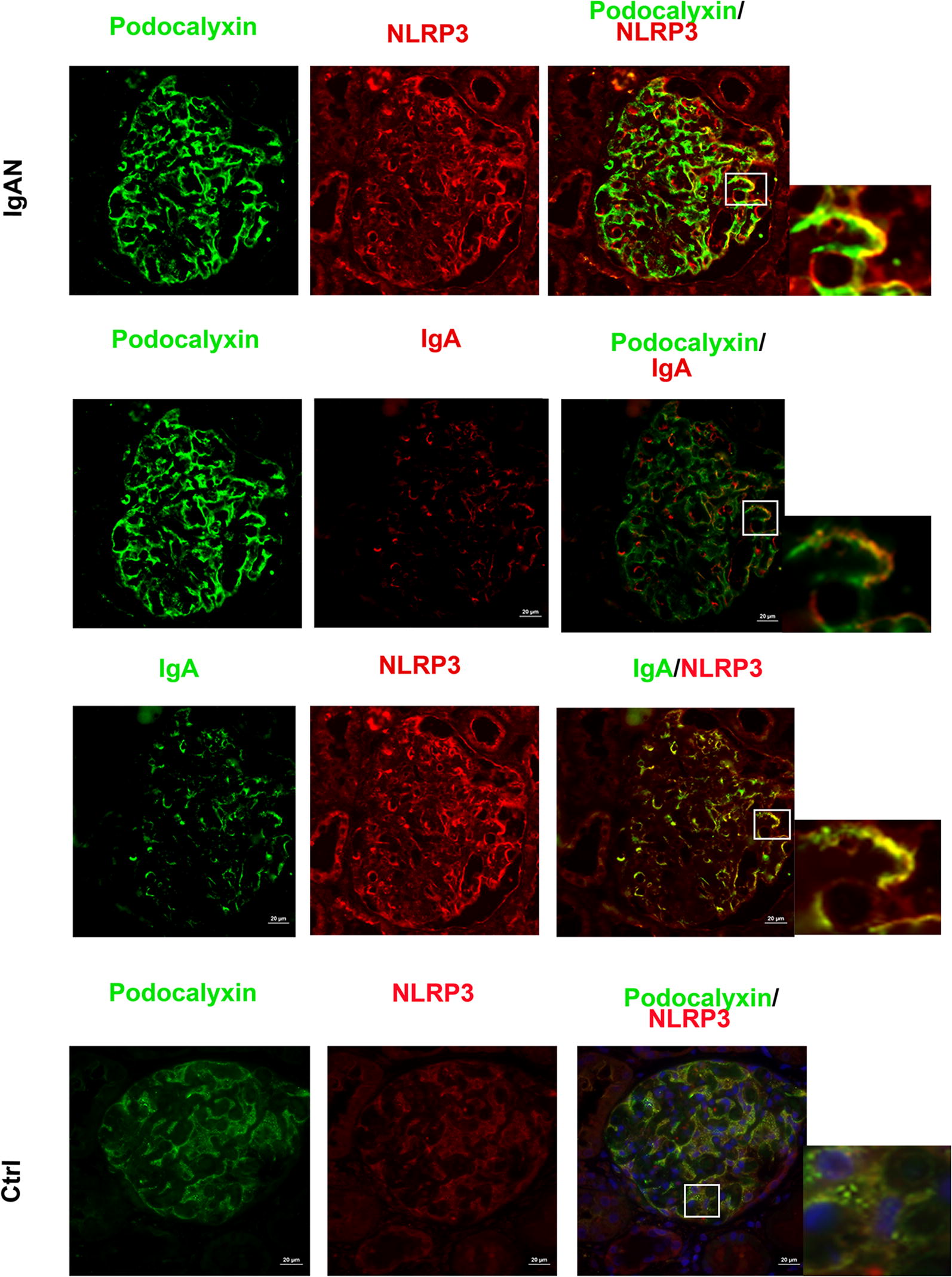
NLRP3 localizes to podocytes in human IgAN. Dual labeling for NLRP3 and either podocalyxin or IgA was performed in biopsy sections. Nuclear staining with DAPI (blue) is shown in merged images. Original magnification: ×400. *IgAN* IgA nephropathy, *Ctrl* control

**Fig. 2:**
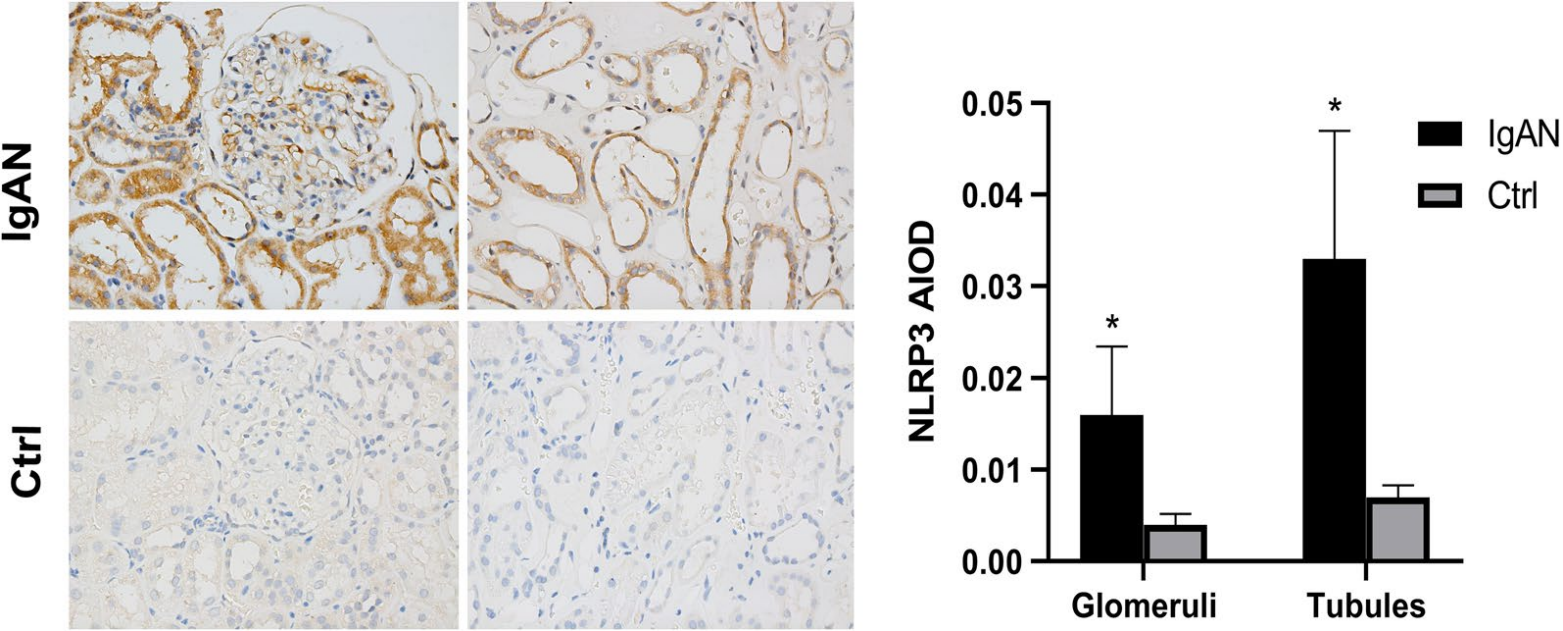
NLRP3 expression in human IgAN. Original magnification: ×400. *IgAN* IgA nephropathy, *Ctrl* control

Immunofluorescent staining analysis revealed that NLRP3, podocalyxin (a major sialoprotein in podocytes) and IgA1 co-localized in the podocytes of IgAN patients. However, NLRP3 staining was not detected in normal control kidney samples (Fig. 1). While deposition of IgA in the glomerular mesangium is a hallmark ofIgAN, our finding suggest that IgA1 may accumulate specifically in podocytes, where it may stimulate NLRP3 expression in IgAN patients. Direct deposition of IgA in IgAN patient podocytes has not been described in the literature previously, so this finding may help provide an oval clue to better understanding the pathogenesis of IgAN. Further immunohistochemistry staining analysis showed that NLRP3 could be detected in the glomeruli and tubules of renal biopsy tissue from IgAN patients, while only very low levels of NLRP3 could be detected in normal renal tissue (Fig. 2). We observed that NLRP3 was significantly increased in the glomeruli (0.016 ± 0.0075 vs 0.004 ± 0.0012, *P *< 0.001) and tubules (0.033 ± 0.0136 vs 0.007 ± 0.0013, *P *< 0.001) IgAN patients compared to controls (Fig. 2).

### Expression of NLRP3 varies with clinical manifestation of IgAN

The association between expression of NLRP3 in glomeruli and tubules and patients’ clinical manifestation of IgAN were analyzed. We found that NLRP3 expression in different regions of the kidney was significantly different in IgAN patients grouped based on clinical features.

**Fig. 3:**
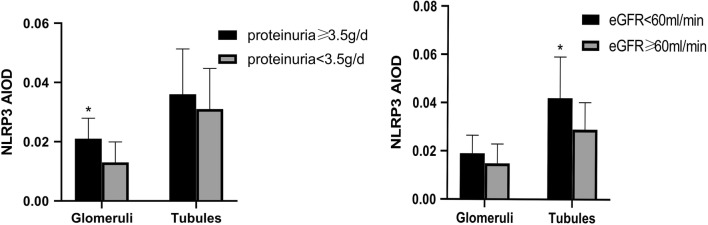
Comparison of glomerular and tubular NLRP3 expression based on the average IOD in patients. Patients were grouped by levels of proteinuria and eGFR. *IOD* integrated optical density, *eGFR* estimated glomerular filtration, *IgAN* IgA nephropathy, *Ctrl* control. *P < 0.05

Significantly higher glomerular NLRP3 levels were detected in patients with more severe proteinuria (≥ 3.5 g/day) compared to patients with less proteinuria (< 3.5 g/day) (0.021 ± 0.0069 vs 0.013 ± 0.0069, *P *= 0.013). However, tubular NLRP3 expression levels were similar between these two groups (0.036 ± 0.0153 vs 0.031 ± 0.0137, *P *= 0.360) (Fig. 3). Significantly higher renal tubular NLRP3 expression levels were detected in patients with severe renal dysfunction (eGFR < 60 ml/min/1.73 m^2^) than in patients with less severe renal dysfunction (eGFR ≥ 60 ml/min/1.73 m^2^) (0.042 ± 0.0171 vs 0.029 ± 0.0112, *P *= 0.034). However, glomerular NLRP3 expression levels were similar between these two groups (0.019 ± 0.0075 vs 0.0149 ± 0.0080, *P *= 0.318) (Fig. 3).

### α-SMA and ICAM-1 expression is increased in glomeruli of IgAN patients

**Fig. 4:**
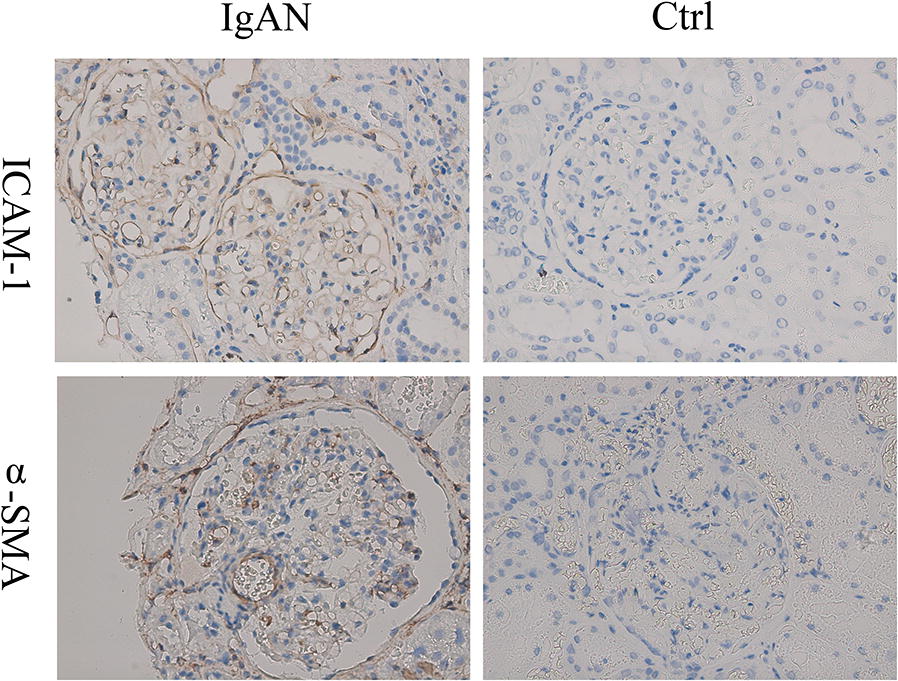
α-SMA and ICAM-1 expression in human IgAN. Original magnification: ×400. *IgAN* IgA nephropathy, *Ctrl* control

α-SMA and ICAM-1 were expressed in the glomeruli of IgAN patients (Fig. 4). Further analysis indicated that the ICAM-1 protein expression level (IHC stain AIOD value) in glomeruli [0.03165 (0.02750–0.04345) vs 0.00004 (0.00002–0.00052), P = 0.009] and the α-SMA protein expression level in glomeruli (0.0255 ± 0.0066 vs 0.003 ± 0.0027, P < 0.001) of IgAN patients were significantly higher than in controls.

### Serum IgA1 may induce PMT in IgAN

**Fig. 5:**
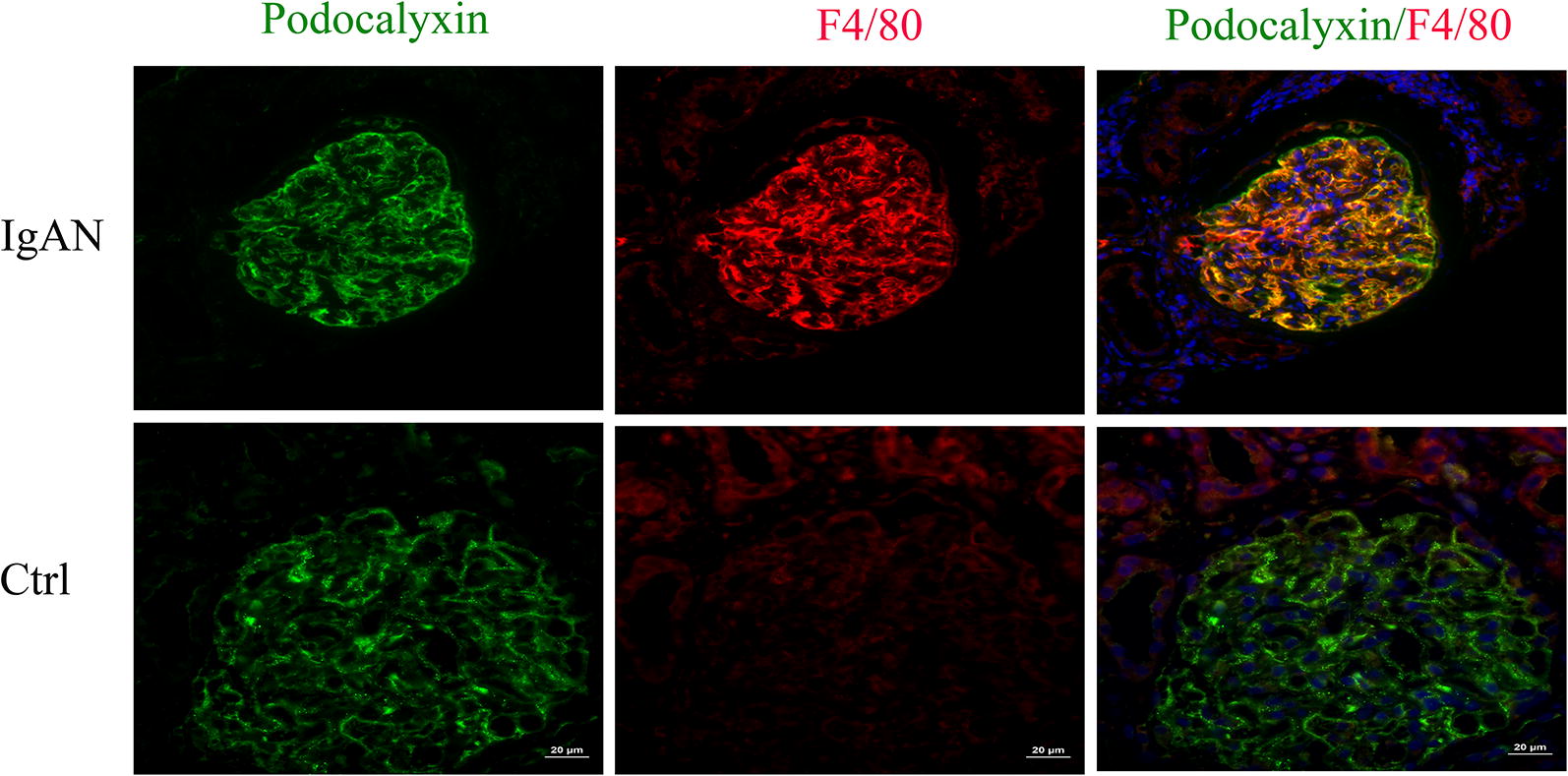
F4/80 localizes to podocytes in human IgAN. Dual labeling for F4/80 (red) and podocalyxin (green) was performed. Nuclear staining with DAPI (blue) is shown in merged images. Original magnification: ×400. *IgAN* IgA nephropathy, *Ctrl* control

**Fig. 6:**
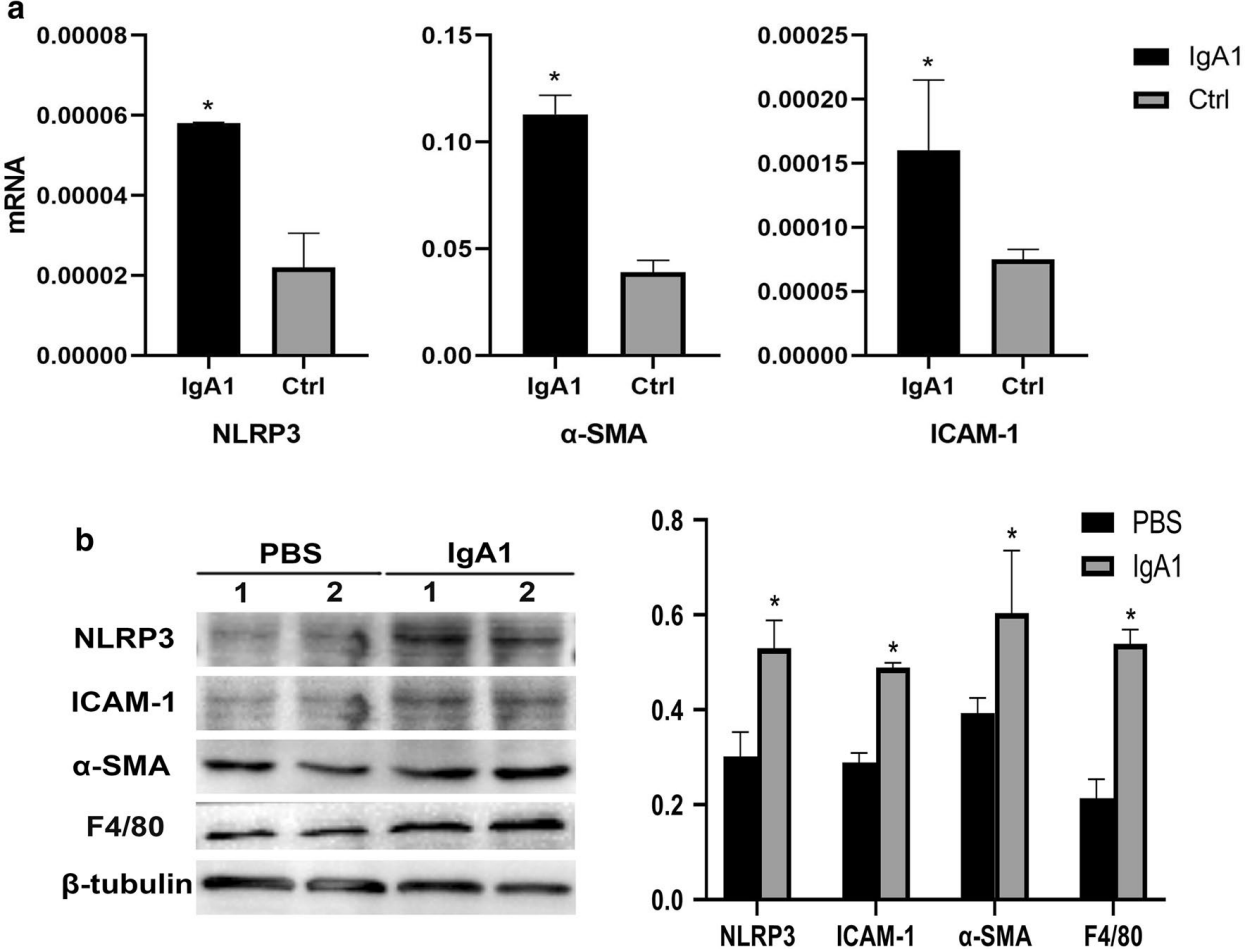
**a** IgA1-stimulated mRNA expression of NLRP3, α-SMA and ICAM-1 mRNA expression in MPC-5 cells. **b** IgA1-stimulated protein expression of NLRP3, α-SMA, ICAM-1 and F4/80 protein expression in MPC-5 cells. *P < 0.05

**Fig. 7:**
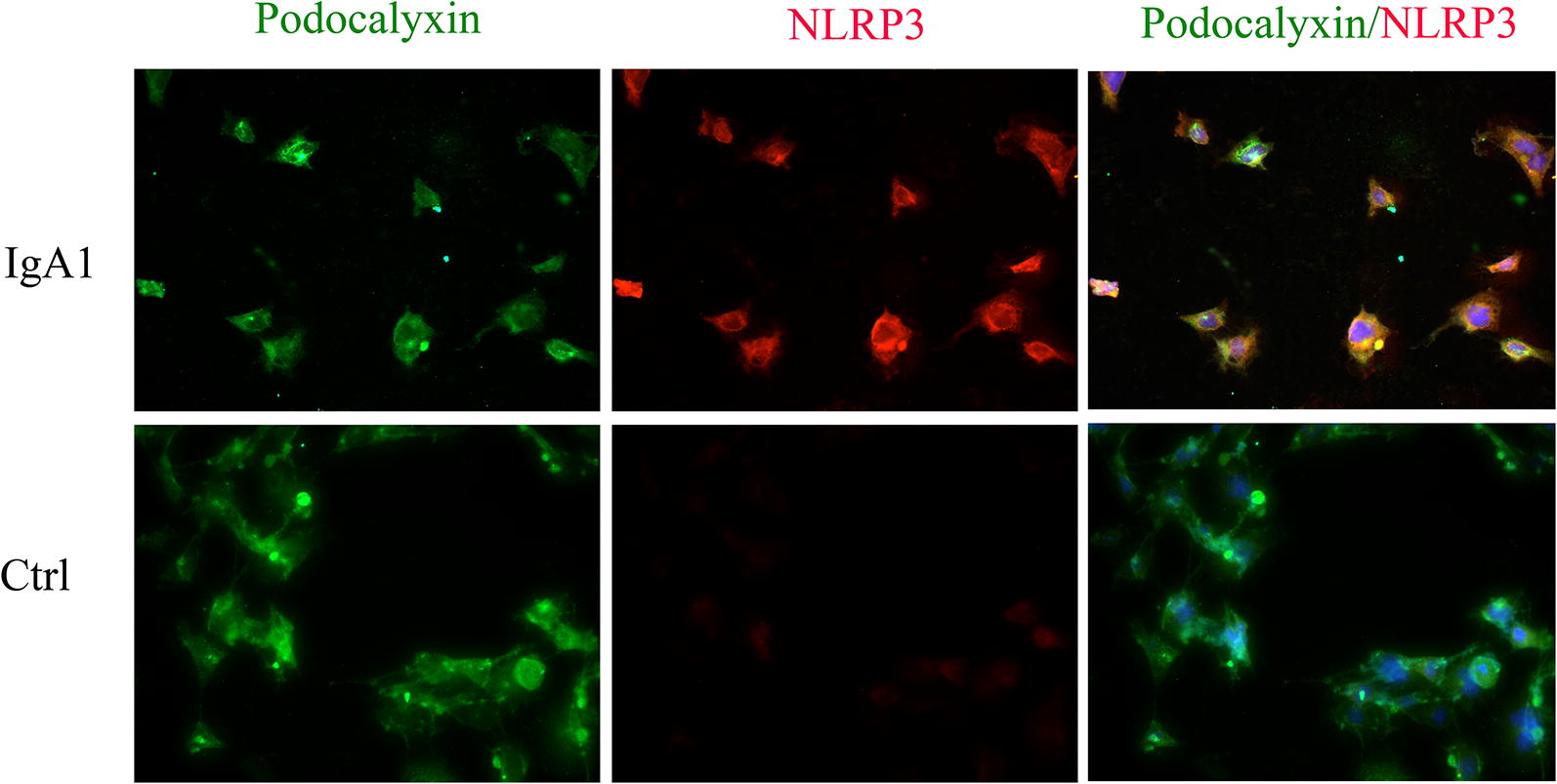
Colocalization of NLRP3 and podocalyxin in MPC-5 cells stimulated by IgA1. Dual labeling of NLRP3 (red) and podocalyxin (green) was performed. Original magnification: ×400

**Fig. 8:**
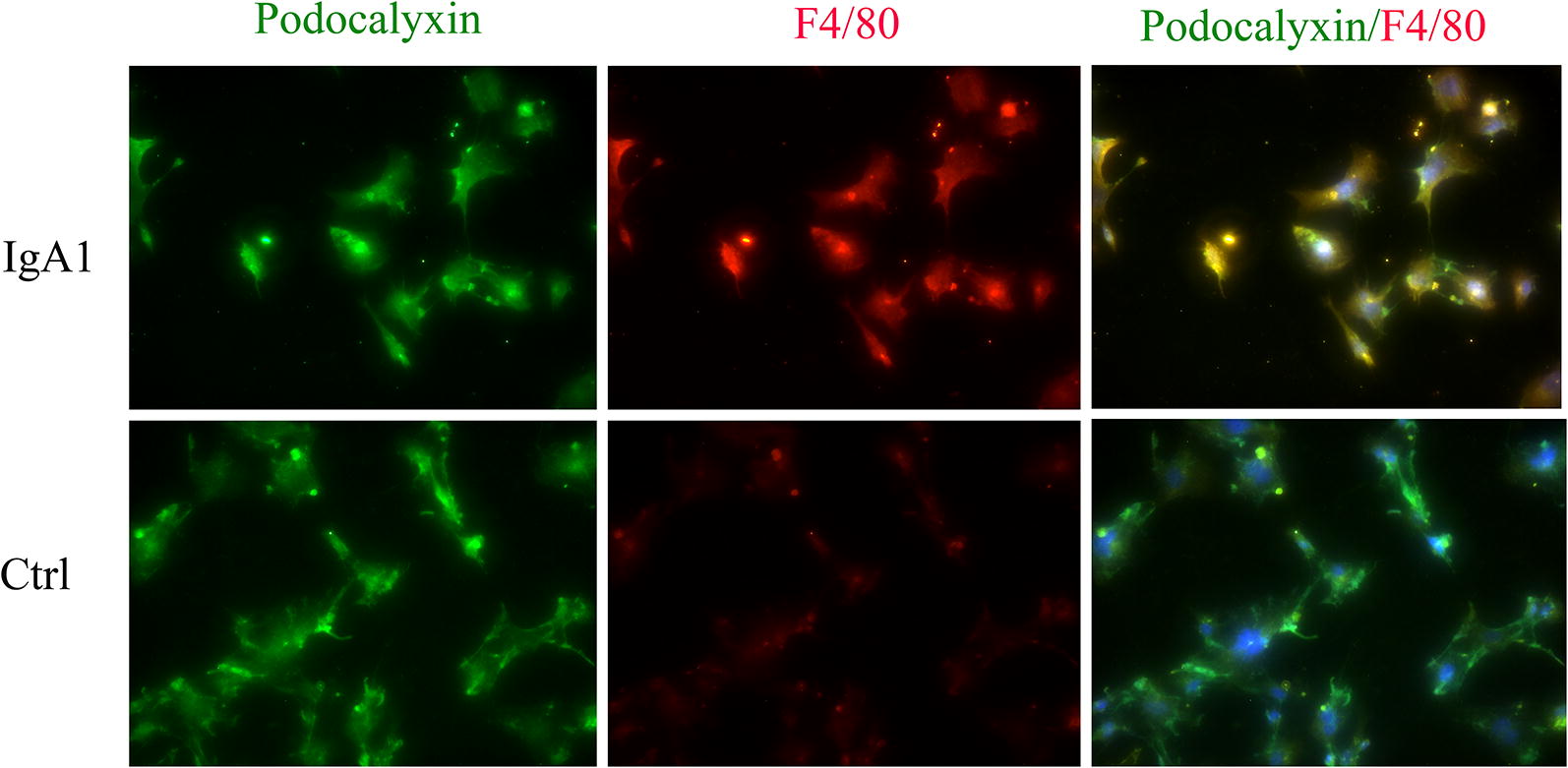
Colocalization of F4/80 and podocalyxin in MPC-5 cells stimulated by IgA1. Dual labeling of F4/80 (red) and podocalyxin (green) was performed. Original magnification: ×400

**Fig. 9:**
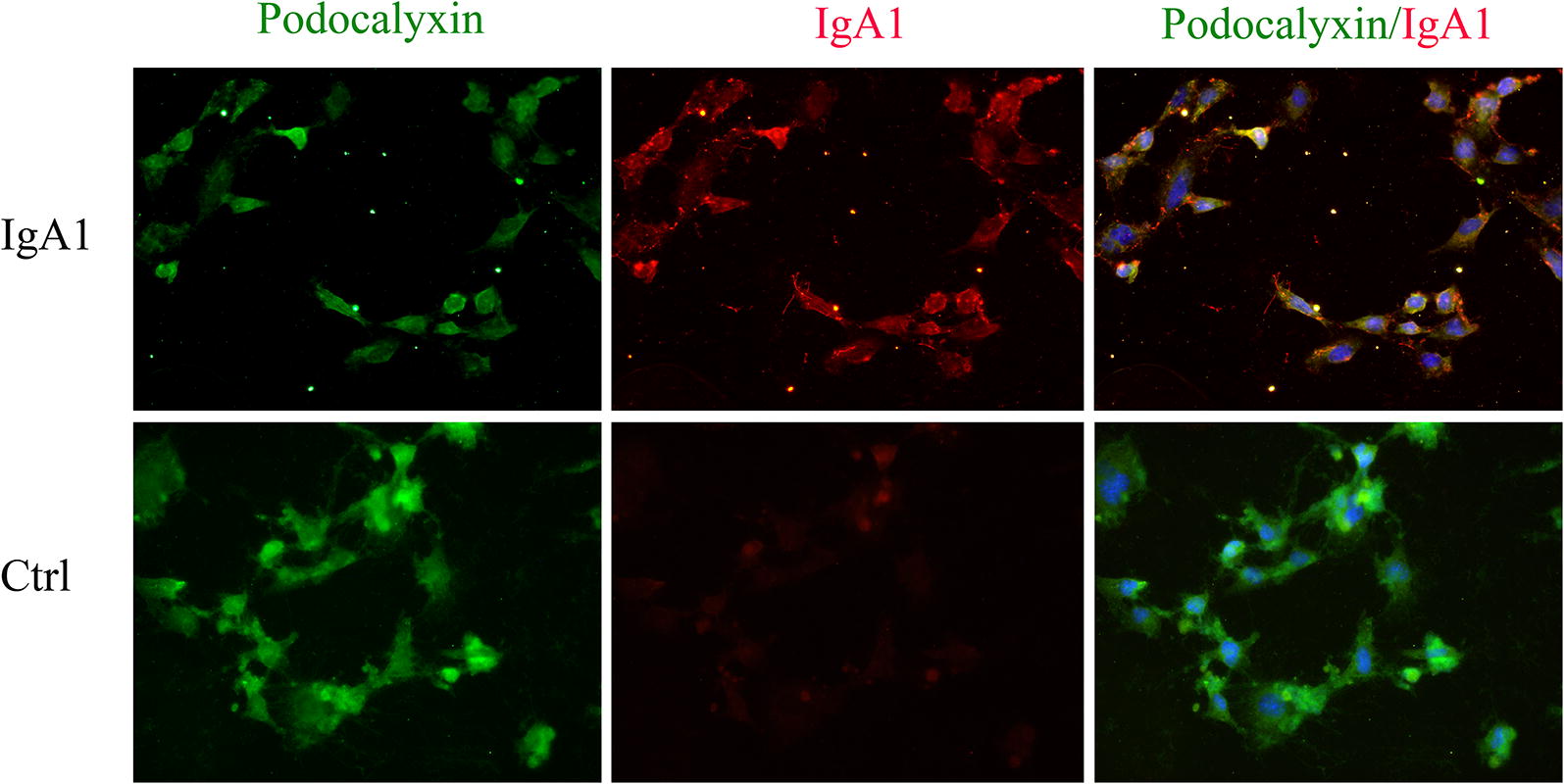
Colocalization of IgA1 and podocalyxin in MPC-5 cells stimulated by IgA1. Dual labeling of IgA1 (red) and podocalyxin (green) was performed. Original magnification: ×400

To examine the expression of macrophage markers in podocytes from IgAN patients, a double staining with an antibody against the podocyte marker podocalyxin and an antibody against the established macrophage marker F4/80 was performed. We observed that F4/80 could be detected in podocalyxin-positive podocytes in biopsy samples from IgAN patients. However, only the podocyte marker and not the macrophage marker could be detected in the control patient biopsy samples (Fig. 5). In order to investigate the role of NLRP3 and F4/80 in podocytes, MPC-5 cells were stimulated with serum IgA1 purified from IgAN patients. After 36 h of stimulation, the mRNA and protein level of NLRP3 and F4/80 increased significantly compared to control (Fig. 6). Immunofluorescent staining also indicated that serum IgA1 stimulation could induce NLRP3 and F4/80 expression in MPC-5 cells (Figs. 7, 8). Co-localization of podocalyxin and IgA1 was present in MPC-5 cells (Fig. 9), indicating that IgA1 accumulated intracellularly. Furthermore, after serum IgA1 stimulation, the protein levels of the inflammatory mediator ICAM-1 and the myofibroblast marker α-SMA increased remarkably compared to unstimulated cells (Fig. 6). These results suggested that serum IgA1 may induce NLRP3 expression and initiate PMT, which could promote increased inflammation and renal fibrosis.

## Discussion

IgAN is the one of the most common causes of primary glomerulonephritis and a leading cause of ESRD. However, the exact pathogenic mechanism underlying IgAN remains largely unknown. It is well accepted that development of proteinuria is a major risk factor for disease progression, and is worsened by diminished podocyte function and survival [13]. Previous studies have indicated that dys-glycosylated IgA1 deposits in the mesangial and para-mesangial area of the glomeruli can induce apoptosis and transdifferentiation of podocytes in IgAN, which in turn leads to dysfunction of the renal filtration barrier and development proteinuria [14]. However, the exact mechanism by which dys-glycosylated IgA1 induces podocyte dysfunction in IgAN is still unknown.

Recently, several studies have suggested that NLRP3 could mediate podocyte dysfunction in several different kidney diseases [15–17]. NLRP3 is a critical regulator of inflammation and is activated upon exposure to pathogens or damage-associated molecular patterns (PAMPs or DAMPs) and environmental irritants [18]. Research focused on understanding the relationship between podocytes and NLRP3 activity may help improve understanding of IgAN pathogenesis and identify novel therapeutic targets. In this study, we found that NLRP3 expression was significantly increased in the glomeruli and tubules in IgAN patient renal biopsy tissues when compared to normal kidney tissues, which is consistent with previous findings [19]. Interestingly, NLRP3 expression levels were found to vary in IgAN patients with different amounts of renal dysfunction and proteinuria. Significantly higher NLRP3 levels were detected in the renal tubules of patients with lower eGFR levels (< 60 ml/min/1.73 m^2^). Moreover, significantly higher NLRP3 levels were detected in the glomeruli of IgAN patients with severe proteinuria (≥ 3.5 g/day). Our findings suggest there is differential expression of NLRP3 based on the clinical manifestation of IgAN, which should be validated by future studies with larger sample sizes. Only one study has previously reported a positive correlation between NLRP3 expression and proteinuria in patients with glomerulonephritis; however, patients with several types of primary glomerular disease were included in that study [6].

Our findings indicated that NLRP3 expression was localized to podocytes in kidney biopsies from IgAN patients. Previously, IgA deposition was known to occur in the mesangial region and occasionally seen in glomerular capillary walls, but was never described in podocytes. We observed co-localization of IgA and podocalyxin, which provides evidence that IgA can accumulate in podocytes. Moreover, we also demonstrated that podocalyxin and IgA1 co-localize in MPC-5 cells. While we did not do experiments to confirm direct binding, the findings in this study provide strong evidence that intracellular IgA1 accumulation in podocytes could account for podocyte phenotypes associated with IgAN. Our in vitro experiments suggest that serum IgA1 isolated from IgAN patients is sufficient to increase NLRP3 expression and pro-inflammatory cytokine expression in podocytes. These results indicate that IgA accumulation in podocytes may induce NLRP3 expression and initiate podocyte injury, transdifferentiation and subsequent inflammatory response, thus promoting the development of proteinuria in IgAN.

For decades, podocytes injury has been seen as a hallmark of glomerulonephritis. Impaired podocyte structure and function leads to proteinuria and renal failure in variety of renal diseases, including diabetic nephropathy, lupus nephritis and membranous nephropathy. However, increasing evidence has indicated that podocytes may actively participate in immune-mediated damage by recruiting inflammatory cells or even acting as antigen-presenting cells or macrophages [20, 21]. It was reported that podocytes may clear immune complexes from the glomerular basement membrane and participate in development of renal inflammation and fibrosis [22]. In this study, we found that the podocytes of IgAN patients could express the macrophage marker F4/80. We also found that serum IgA1 from IgAN patients could stimulate the expression of NLRP3 and F4/80 in MPC-5 cells, which resulted in increased expression of the inflammatory mediator ICAM-1 and the myofibroblast marker α-SMA. The expression of α-SMA and ICAM-1 was also significantly higher in the glomeruli of IgAN patients, indicating the occurrence of mesenchymal transdifferentiation and induction of inflammation. These observations suggest that podocytes stimulated with dys-glycosylated IgA1 may acquire macrophage-like functions and undergo transdifferentiation into inflammatory cells that release pro-inflammatory cytokines and participate in pathological changes that drive IgAN. Unfortunately, our present study is unable to implicate the specific role of NLRP3 which might be played in driving PMT. PMT is relatively new concept that is indicated by some findings in this preliminary study. We intend to carry out further research in transgenic mice to validate the hypotheses about PMT and IgAN generated by the findings in this study. Previous investigations have shown that NLRP3 can be activated in macrophages and regulate their function [23]. The increased NLRP3 expression described in the present study could be the result of PMT or some other aspect of IgAN pathogenesis. Whether NLRP3 activation and expression is involved in podocyte transdifferentiation in the context of IgAN needs to be evaluated in future studies.

Our observations provide new evidence for a novel and unexpected role of podocytes in IgAN pathogenesis. Based on our findings, we hypothesize that in the context of IgAN, serum dys-glycosylated IgA1 may stimulate podocytes directly and induce the expression of NLRP3, which will initiate PMT. As a result of PMT, podocytes will acquire macrophage-like characteristics and release inflammatory cytokines, which will promote inflammation and renal fibrosis. The current study is provides evidence to support this hypothesis, but further study is required for validation.

## Conclusion

In conclusion dys-glycosylated IgA1 isolated from IgAN patient serum can induce NLRP3 expression in podocytes, which may initiate podocyte macrophage transdifferentiation (PMT). After PMT, podocyte scan secrete proinflammatory cytokines that could contribute to promoting the inflammation cascade and renal fibrosis associated with IgAN.

## Data Availability

All data generated or analyzed during this study are included in this article.

## Abbreviations

IgAN: immunoglobin A nephropathy
ESRD: end-stage renal disease
NLRP3: NOD-like receptor, pyrin domain-containing 3
eGFR: estimated glomerular filtration rate
CKD-EPI: chronic kidney disease epidemiology collaboration
IOD: integrated optical density
PMT: podocyte macrophage transdifferentiation
